# Draft Genome Sequence of a Mycobacterium porcinum Strain Isolated from a Pet Cat with Atypical Mycobacterial Panniculitis

**DOI:** 10.1128/MRA.00006-20

**Published:** 2020-03-12

**Authors:** Anthony Mannion, Tina McCollester, Alexander Sheh, Zeli Shen, Hilda Holcombe, James G. Fox

**Affiliations:** a Division of Comparative Medicine, Massachusetts Institute of Technology, Cambridge, Massachusetts, USA; b Veterinary Practice, New Bedford, Massachusetts, USA; Loyola University Chicago

## Abstract

A fast-growing *Mycobacterium* species was cultured from draining, purulent lesions on the caudal abdomen of a 12-year-old male domestic long-haired cat. Whole-genome sequencing identified the organism as Mycobacterium porcinum.

## ANNOUNCEMENT

A 12-year-old male domestic cat presented with multiple purulent, draining lacerations on the caudal abdomen that failed to resolve with amoxicillin-clavulanic acid (Clavamox; Zoetus, Parsippany, NJ) (62.5 mg twice daily) and pradofloxacin (Veraflox; Bayer, Shawnee Mission, KS) (7.5 mg/kg of body weight twice daily) treatment. Histological analysis of surgically excised lesions 8 weeks after initial presentation identified multifocal, coalescing inflammatory infiltrates associated with granulation tissue and small cavitated spaces containing acid-fast-negative, Gram-positive bacilli. Skin lesions did not completely resolve despite protracted multiantibiotic treatment. Eighteen months after initial presentation, a fast-growing mycobacterium was cultured from full-thickness biopsy specimens of remaining lesions using sheep blood agar incubated for 5 days at 37°C (IDEXX Laboratories, North Grafton, MA). Other organisms were not cultured. The isolate was transferred to the Jewish Medical and Research Center (Denver, CO) and grown on 7H11 medium at 32°C under aerobic conditions with 5% CO_2_ for culture and antibiotic sensitivity testing. The isolate exhibited resistance to ceftriaxone, cefipime, cefotaxime, imipenem, trimethoprim-sulfamethoxazole, and amoxicillin-clavulanic acid and intermediate resistance to cefoxitin, doxycycline, minocycline, azithromycin, and linezolid. The mycobacterium may have been part of a polymicrobial infection that was undetected by initial acid-fast staining or was a subsequently acquired infection. A diagnosis of atypical mycobacterial panniculitis was made based on culture results. A bacterial pellet of the mycobacterium isolate was sent to the Massachusetts Institute of Technology for DNA extraction, whole-genome sequencing, and bioinformatics analyses (default parameters were used unless stated otherwise). The cat was euthanized due to deteriorating health.

DNA was purified using the High Pure PCR product purification kit (Roche Laboratories, Indianapolis, IN). Barcoded libraries were constructed using the QIAseq FX DNA library kit (Qiagen, Germantown, MD) and sequenced using Illumina MiSeq 2 × 300-bp reads. Raw sequence reads were decontaminated of adapters and quality trimmed using BBDuk v38.34 (with the parameters ktrim=r, k=23, mink=11, hdist=1, tpe, tbo, qtrim=rl, trimq=10, and qin=33), resulting in 1,286,206 raw reads. SPAdes v3.10.0 hosted by PATRIC (1) was used for *de novo* sequencing. The resulting draft genome had 6,796,350 bp in 72 contigs with a coverage of 35.45×, *N*_50_ value of 246,450 bp, and GC content of 66.8%. Annotation using PGAP v4.11 (2) identified 6,523 proteins, 53 tRNAs, and 10 rRNA genes.

The genome was most closely related to those of Mycobacterium porcinum strains as determined using JSpeciesWS (3) and whole-genome phylogenetics using the Bacterial Pan Genome Analysis (BPGA) tool v1.3.0 (4). The mycobacterium genome had average nucleotide identities (3) of >95% and digital DNA-DNA hybridization (5) percentages of >70% compared to other M. porcinum genomes (GenBank accession numbers OLMG01000000, MIHF01000000, MVIG00000000, MBDY00000000, MBEH00000000, MBEF00000000, CCBG00000000, and MBEG00000000). Antibiotic resistance genes to streptogramin [*erm*(39)], lincosamide [*erm*(39)], macrolides [*erm*(39) and *mtrA*], penam (*mtrA*), fosfomycin (*murA*), tetracycline (*tap*), rifamycin (*rbpA* and *aar-1*), and aminoglycosides [*aac(2′)-Ib*] were predicted using Resistance Gene Identifier (RGI) v5.1.0 (6) and were consistent with the antibiotic phenotype of the M. porcinum isolate from the cat. Virulence factor genes, including the type VII secretion systems ESX-1, ESX-3, and ESX-4 (7, 8), mammalian cell entry operons (9, 10), glycopeptidolipid biosynthesis (9), the iron siderophore mycobactin, fibronectin binding protein (9), and 19-kDa lipoprotein antigen (9), were predicted using VFanalyzer (11).

M. porcinum was originally isolated from pigs with lymphadenitis and since then has been detected in domestic and wild animals as well as drinking water, vegetables, and cow milk (12). M. porcinum is an opportunistic pathogen reported to cause wound infection, respiratory infection, bacteremia, and postoperative infection (12). This organism has not been previously associated with feline atypical mycobacterial panniculitis. Inconsistencies regarding published biochemical and genetic characteristics have made the identification of *Mycobacterium* species challenging. For example, several M. porcinum genomes have been repeatedly misidentified/mislabeled as Mycobacterium vulneris (13). Accurate genome sequencing and reporting is essential because of the zoonotic potential of *Mycobacterium* species.

### Data availability.

This genome sequence has been deposited in GenBank under the accession number VNFN00000000. The sequencing reads have been deposited in the SRA under the accession number SRR9831353.
